# Does Post-Traumatic Growth Follow Parental Death in Adulthood? An Empirical Investigation

**DOI:** 10.1177/0030222820961956

**Published:** 2020-09-24

**Authors:** Komal Qasim, Jerome Carson

**Affiliations:** 1 University of Bolton, Bolton, UK

**Keywords:** post-traumatic growth, grief, adulthood, bereavement, Covid-19

## Abstract

This study looked at the loss of a parent in adulthood and whether this was followed by post-traumatic growth? Participants, 100 bereaved adults, from Pakistan and England, lost parents in the last 10 years. They completed three questionnaires. The study hypotheses were, first, that participants whose bereavement occurred more than five years ago would show significantly higher levels of post-traumatic growth. Second, participants with higher levels of post-traumatic growth would experience significantly higher grief scores. Thirdly, participants with higher levels of post-traumatic growth would show significantly higher levels of coping skills. Two hypotheses were rejected, only one received partial support. Yet, levels of post-traumatic growth were high in this sample. Post-traumatic growth does not follow every bereavement. The authors provide autoethnographic material to challenge this. Circumstances surrounding bereavement during the Covid-19 pandemic, are more likely to lead to increases in complicated grief reactions, rather than post-traumatic growth.

## Introduction

Death is inevitable and may have serious physical and psychological implications for the survivor (Crunk, 2017). Experiencing intense grief, mourning, loss and bereavement are universal experiences following death, but are unique to each individual. In spite of the fact that every individual experiences and feels the pain of grief differently, there is still a mutual and common experience of loss (Trauthwein, 2015). In this respect, the unique way in which every individual responds to death has been of psychological interest for hundreds of years (Granek, 2010). In the context of grief and bereavement, Abrams (2000) stated that parental death is one of the most painful incidents in an individual’s life, which has both emotional and psychological effects on individuals, regardless of their age. Furthermore, the death of a parent, multiple deaths and traumatic death, may lead to complicated grief reactions (Tobin et al., 2020). Several researchers have highlighted the significance of parental bereavement during adulthood and the fact that this subject has not been well researched in the scholarly literature (Koblenz, 2015; LaFreniere & Cain, 2015). Moreover, Harris (1991), suggested that young adults are the major group of individuals who find it difficult to express their true feelings to family and friends. This makes them a vulnerable and significant group to investigate, as not as much research has been conducted to find the impact of parental death on adults (Glass, 1990).

In recent years a new field has developed in Psychology, which has been named positive psychology. It is based on a model of strengths, growth and well-being, whose main goal is to promote human flourishing (Seligman, 2002; Seligman & Csikszentmihalyi, 2000). Post-traumatic growth is a construct that has been investigated in positive psychology, which focuses on the positive psychological changes in an individual following trauma (Tedeschi & Calhoun, 2004). Experiencing any growth following grief, does not mean that the negative effects of pain and suffering are not dealt with following loss. On the other hand, it is predicted and expected, that post-traumatic growth should actually reduce psychological distress (Calhoun et al., 2010). Positive psychology tries to focus on the positive outcomes of traumatic experiences (Calhoun & Tedeschi, 1998). In other words, the very experience of loss can sometimes lead to increased strength and more self confidence in some individuals (Calhoun & Tedeschi, 1990; Carnelley et al., 2006).This phenomenon named as post-traumatic growth by Tedeschi and Calhoun (2004), is defined as a positive modification or a transition of an individual, which follows challenging life events. In this respect, it can be postulated that finding meaning from the death of a loved one might be a healthy way of recovering on the part of the bereaved individual and may help them grow psychologically and emotionally (Fisher & Specht, 1999). According to a study by Armstrong and Shakespeare-Finch (2011), higher levels of post-traumatic growth were reported by individuals who had lost their first degree relative (parent, sibling, child), as compared to those participants who had lost a second or third degree relative (cousin or a friend). The notion that something positive can come from the death of a loved one, may be totally unacceptable for some and even disturbing for others, as the pain of the loss is too intense (Calhoun & Tedeschi, 2001). Generally speaking, when any negative and traumatic event occurs, especially death, the first reaction to the trauma is negative emotion i.e. post traumatic fear and shock (that may turn into post-traumatic stress disorder or PTSD).

The purpose of this paper is to examine the phenomenon of post-traumatic growth, following the death of a parent in adulthood. The rationale behind studying post-traumatic growth, grief and coping strategies amongst bereaving adults, is that the strong bond between parents and children when disrupted by the harsh reality of death, results in the loss of the source of comfort on the part of the bereaving adults, which may cause a decline in their subjective well-being (Umberson, 2003). Getting over the death of a loved one and moving on is not an easy task, especially losing a parent. It is a life changing event in any individual’s life, which raises the question as to whether post traumatic growth really occurs after the loss of a loved one or not? Also, the various coping strategies used by bereaving adults need to be explored. Every individual has a different way of coping with loss (Apelian & Nesteruk, 2017; Bonanno, 2004). Matthews (2006) noted therefore that the way they may find meaning in their lives and try to cope with the grief, is also different. The well-being of bereaving individuals is affected by their unique coping styles and the way they adapt to their parental death (Howell et al., 2015). Helgeson et al. (2006) found inconsistent data pertaining to the relationship between measures of psychological distress and growth. Growth still tends to be a statistically independent construct like post-traumatic loss is (Baker et al., 2008; Cann et al., 2010).However not all individuals who experience some form of loss will exhibit post traumatic growth. Therefore, Calhoun and Tedeschi (1998) made an effort to shift the focus from the negative impact of loss to a more positive outcome, thus potentially preventing post-traumatic stress disorder. However, no significant relationship was found between complicated grief symptoms and post traumatic growth amongst bereaving youth (Salloum et al., 2019).

The death of a parent can also have a significant influence on the way the individual views themselves and the world (Umberson, 2003). A major reason for this anguish is that adulthood is a transitional period in which adults may only have started to live independently from their own parents in terms of employment, higher education and marriage (Austrian, 2008). MacCallum et al. (2017) reported that grief following death is a collective experience of emotional, behavioural, motivational and cognitive reactions. It may involve missing the deceased intensely, a lack of acceptance of the death on the part of the bereaved, a self-identity crisis, confusion, numbness, bitterness, losing trust and an impossibility of getting re-involved with life and relationships again. However, the intensity with which every individual experiences the above mentioned reactions, varies from person to person (Maccallum et al., 2017).

Interestingly, if in contrast to the negative effects following trauma, positive outcomes are considered, then generally positivity following trauma means recovering and coping with the loss. It may also mean coming back to the previous state of mind that the individual may have had prior to the adversity or the traumatic event. Post-traumatic growth however means not only returning to the prior state, but rather emerging as a better and stronger person in an improved psychological state, than the individual was before experiencing the trauma. This may be the result of the struggle that the person makes in order to thrive through adversity (Calhoun & Tedeschi, 1998; Christopher, 2004; Linley & Joseph, 2004; Tedeschi & Calhoun, 2004).The pain of losing a loved one may help people to redefine their relationships and treasure what they are left with (Pollock, 1982). Research suggests that for some individuals, coping with grief is a very complicated process (Prigerson et al., 2009; Shear et al., 2011). With others, grief may subside within two years (Bonanno, 2004).

It is undeniable that the loss of a loved one will certainly cause varying degrees of distress and negative thoughts. But studies reveal that with the passage of time, the grieving person might begin to direct their activities and thoughts towards more constructive and goal directed behaviours. For some people, positivity is approached after the immediate shock and distress, but for others, both behaviours may coexist (Stroebe & Schut, 1999). Calhoun et al. (2010), also suggested that conscious contemplation towards positivity may help the bereaved individual to gradually rebuild the shattered image of their world and discover meaning in death. On the other hand the inability to do so might result in continued stress and prolonged grief. A study done by McClatchey (2018), suggested that the circumstances of parental death can also predict post traumatic growth in bereaving youth, claiming that sudden parental death leads to higher levels of PTG, than an expected parental death.

The year 2020 has seen widespread changes in the world in terms of the Covid-19 Pandemic and the possibility of complicated grief following parental death seems to be greater than that of post-traumatic growth. A recent study by Wallace et al. (2020), postulated that the Covid-19 pandemic would lead to higher levels of complicated and anticipatory grief for the effected and bereaving family members, individuals and health providers. The fact that funerals and burials are either held distantly or suspended altogether, has further led to the possibility of complicated grief reactions amongst the bereaving individuals. Furthermore, the entire concept of bereavement and grief has been altered following the sociological, emotional and financial impact of Covid-19, that the world has seen in 2020. Since social distancing has been implemented by almost every country in the world, this has resulted in hospitals not allowing access to the family members of the dying loved ones. Therefore, in the case of any death, the possibility of complicated grief as a result of physical, mental and social distancing will be much higher (Wallace et al., 2020). The severity of grief resulting in complicated grief symptoms could also be increased among those bereaving individuals, who lost a family member as a result of any other cause except Covid-19, as government policies towards tackling the pandemic, means they have also been affected (Eisma et al., 2020).

The present study had the following hypotheses:

1. Study participants whose bereavement occurred more than five years ago will have significantly higher levels of post-traumatic growth on the Post-Traumatic Growth Inventory.
2. Participants with higher post-traumatic growth will experience significantly lower Adult Attitude Towards Grief scores.
3. Participants with higher post-traumatic growth scores on the Post-Traumatic Growth Inventory will have significantly higher scores on the CABLE Coping Skills Scale.

## Method and Participants

The current study was an effort to examine post-traumatic growth and grief following parental death in adulthood. A non-experimental, quantitative and cross-sectional survey method was utilised, to examine individuals who had experienced the death of one or both parents. The study also involved a diverse international sample of bereaving adults, largely from the United Kingdom and Pakistan, targeting the adult population who were grieving the death of one or both parents, within the last 10 years. The sample was limited to participants who were above the age of 18. A convenience sampling method was used in order to collect data, however no power analysis was performed. A total sample of 100 grieving adults was obtained. The rationale behind selecting 100 individuals is that research indicates that a sample size between 30 and 500 at the 5% confidence level is considered sufficient (Altunışık et al., 2004). The proposal received ethical approval from the University Psychology Department ethics committee, in line with the ethical guidelines of the British Psychological Society (2018). No data were collected until ethical clearance was obtained.

### Procedure for Data Collection

Participation in the study was completely voluntary. For the data collection in the United Kingdom, the researcher used personal contacts and went to a local church. With the help of a local priest, data were collected from the Christian community. For the purpose of data collection in Pakistan, the researcher travelled to Pakistan to get the questionnaires filled out in person. In order to make data collection more convenient and quicker, the researcher used Google forms and consolidated all three questionnaires with the demographic items into one complete form. That form was forwarded to friends and family all over the world via email and WhatsApp.

### Measures

As the study consisted of three main dependent variables namely grief, post-traumatic growth and the coping mechanisms of bereaving adults, three questionnaires were used to assess these.

#### Post-Traumatic Growth Inventory

To measure the post-traumatic growth of bereaving individuals following parental loss, the Post-traumatic Growth Inventory of Tedeschi and Calhoun (1996), was administered to participants. This 21-item scale includes five factors; new possibilities; relating to others; personal strength; spiritual change; and appreciation of life. The PTGI is rated on a frequency of occurrence basis, with options ranging from 0-5, with 0 being *‘I did not experience this change at all’* to 5 being *‘a very great degree as a result of my crisis’*. Sample items are, “After the bereavement, *“I developed new interests,”* and *“I can better appreciate each day.”* Tedeschi and Calhoun (1996) reported the internal consistency and test retest reliability of the inventory to be *α* = 0.90. Cronbach’s alpha for the subscales was, ‘new possibilities’ *α* = .84, ‘relating to others’ *α* = .85, ‘personal strength’ *α* = .72, ‘spiritual change’ *α* = .72 and ‘appreciation of life’ *α* = .67 (Tedeschi & Calhoun, 1996).

For the purpose of the present study, and as this study used quite a different sample from that of Tedeschi and Calhoun, the internal reliability of the post-traumatic growth inventory was examined for this population. The overall scale exhibited very good reliability, Cronbach’s alpha (*α* = 0.89). Similarly, all the five subscales of PTGI were checked for internal reliability and consistency and yielded satisfactory psychometric properties. The first subscale of PTGI namely “relating to others” had a reliability of (*α* = 0.79), “new Possibilities” (*α* = 0.79), “personal strength” (*α* = 0.70), “spiritual strength” (*α* = 0.70), and finally the internal reliability of the last subscale, namely “appreciation of life,” was found to be (*α* = 0.60). These are all consistent with the alpha values given by Tedeschi and Calhoun.

#### Adult Attitude to Grief Scale

The Adult Attitude to Grief Scale (Machin, 2007), is a short 9 item validated tool and covers individuals’ coping responses and reactions to loss. This was used to measure the grief responses of individuals in terms of coping. It has three subscales, Resilience, (eg. *“I feel very aware of my inner strength when faced with grief”*), Controlled (eg. “*For me it is important to keep my grief under control”*) and Overwhelmed (eg. *“I feel I will always carry the pain of grief with me”*). It assesses feelings regarding grief. Statements are rated on a five-point Likert scale with options ranging from ‘strongly agree’ to ‘strongly disagree’, (4 to 0). The significance of the Adult Attitude to Grief Scale is that it is now widely and predominantly used as an assessment tool in order to improve therapeutic conversations (Machin, 2007). It also provides an individual grief profile on the basis of the three distinct subscales of this scale namely ‘overwhelmed’, ‘controlled’ and ‘resilience’ (Machin, 2007; Machin & Spall, 2004). Additionally, this scale has a Vulnerability category. Scores of more than 24 represent “severe vulnerability,” scores of 21 to 23 reflect high vulnerability, and scores of lower than 20 reflect low vulnerability.

The scale has acceptable internal consistency, where “overwhelmed” was found to have the highest internal consistency of all three subscales. The mean scores and standard deviation for the three subscales were; “overwhelmed” mean = 8.92 (sd = 2.53) respectively, “controlled” mean = 7.98 (sd = 2.31) and “resilient” mean = 5.25 (sd = 2.46). The mean score and standard deviation for the overall AAG scale was 22.15 (sd = 4.38), (Sim et al., 2014).

In the current study, the internal reliability of the Adult Attitude to Grief scale was determined by Cronbach’s Alpha (*α*). Each of the subscales of the AAG were tested. The reliability of the ‘Resilience’ subscale was found to be *α* = 0.52. The reliability of the ‘overwhelmed’ subscale was *α* = 0.60 and the ‘controlled’ subscale was *α* = 0.81.

#### The Coping Assessment for Bereavement and Loss Experiences (CABLE)

This psychometric scale was developed by Crunk (2017) to identify the coping strategies used by bereaving individuals in order to cope with the grief and pain they experienced as a result of a death of their loved one. It is a 28-item frequency of occurrence scale with response options ranging from 0–5, where 0 being ‘Never’ and 5 being ‘Daily’. There was a neutral option as well i.e. (*N/A – This does not apply to me or to my loss*). Sample items include, *“I reviewed photos or videos of my loved one,” “I talked to my loved one in my mind or out loud,”* and *“I engaged in an act of kindness towards someone.”* The internal consistency calculated by Cronbach’s alpha (*α*) of this scale was *α* = .95. Good internal consistency was also found among the six factors. Help-Seeking *α* = .85; Positive Outlook *α* = .77; Spiritual Support *α* = .86; Compassionate Outreach *α* = .69, Continuing Bonds *α* = .79; and Social Support *α* = .75.

To examine the cross validation and reliability of the CABLE, Cronbach’s alpha (*α*) in the present study was calculated for each of the subscales. Total internal consistency of the 28 items was found to be *α* = .89. Help seeking yielded *α* = .84; Positive Outlook, *α* = .78; Spiritual Support, *α* = .86; Compassionate Outreach, *α* = .66, Continuing Bonds, *α* = .76; and Social Support, *α* = .74.

## Results

For data analysis, SPSS version 23 was used. Both descriptive and inferential statistics were carried out. The demographic variables recorded in the current study were; age of participant, gender, education, marital status, ethnicity, religion, how often they practiced religion, their parent’s age at the time of death, number of years since the parent passed away, cause of parental death and how often the participants were in contact with their deceased parent before their death? (see Table 1). Prior to statistical analysis the three main dependent variables were tested on the Kolmogorov Smirnov Test for normality. Two were found not to be normally distributed. Skewness statistics also showed that two variables had a skewed distribution. The decision was taken to use non-parametric tests for the inferential statistics.

**Table 1.** Demographic Features of the Participants.

Total sample (n=100)	N
Gender	
Male	41
Female	59
Ethnicity	
** **Pakistani	39
** **British	44
American	3
African	14
Other	0
Marital status	
Married	56
Single	40
In a relationship	1
Widowed	2
Divorced	1
Religion	
Christianity	46
Islam	52
Judaism	1
Buddhism	1
Hinduism	0
Atheist	0
Other	0
Age of the participants (M = 34.8 years; SD = 7.6)	
22–35	50
36–51	50
Highest level of education	
Some high school (less than 12 years of education)	3
High school graduate (12 years of education)	27
Completion of university or trade school	23
Some post-graduate or professional school	27
Completed post-graduate or professional degree	20
Engage in religious activities	
Never	7
Once per year	6
At least twice per year	4
At least once per month	6
At least twice per month	5
At least once per week	27
At least twice per week	15
Everyday	30
Parent’s age when passed away (M=64.7; SD = 12.3)	
** **39–64	54
** **65–96	46
Time since parent’s death (M = 5.4; SD = 2.6)	
1–5	47
5–10	53
Cause of parental death	
** **Natural anticipated death	38
** **Natural sudden death	46
Fatal accident	4
Medical malpractice	2
Other	10
Contact with parent before their death	
Everyday	48
2–7 times per week	33
Once per week	10
Every other week	8
Once per month	1

Prior to examining the three study hypotheses, **descriptive statistics** will be presented on the Post-traumatic Growth Inventory, as post-traumatic growth is the main focus of this paper.

The top five rated individual items out of the 21 items on the Scale were as follows:

1. *“I have a stronger religious faith.”* Mean score = 3.57.
2. *“I have a better understanding of spiritual matters.”* Mean = 3.54.
3. *“I discovered that I’m stronger than I thought I was.”* Mean = 3.47.
4. *“I am more likely to try to change things which need changing.*” Mean = 3.40.
5. *“I put more effort into my relationships.”* Mean = 3.36.

The mean item scores per factor were as follows (Cardiac group as a comparison in parentheses):

1. Spiritual Change. Mean score = 3.55. (Cardiac patients = 1.79)
2. Personal strength. Mean score = 3.27. (Cardiac patients = 2.68)
3. Appreciation of life. Mean score = 3.03. (Cardiac patients = 3.39)
4. Relating to others. Mean score = 2.95. (Cardiac patients = 2.90)
5. New possibilities. Mean score = 2.89. (Cardiac patients = 2.41)

**Total PTGI** for current **bereaved** sample score = **64.36 (sd = 16.44)**

Total PTGI for **Cardiac group** (n = 124) = **56.84 (sd = 24.19)**

(Sheikh & Marotta, 2005).

The current sample are compared with a cardiac sample simply to give an idea of the level of post traumatic growth in the present sample, which is higher than in the cardiac sample and also to show the different factor scores. For the present sample spiritual factors were clearly very important, with the two items making up this factor gaining the highest individual scores of all PTGI items.

Three hypotheses were tested in the study. Each of these will be examined in turn.

1. **Study participants whose bereavement occurred more than five years ago will have significantly higher levels of post-traumatic growth on the Post-Traumatic Growth Inventory.**

To test this hypothesis, the current sample was divided into those who had lost a parent in the last five years (n = 53), and those whose parent had died more than five years ago (n = 47) (see Table 2). It seems reasonable to assume that post-traumatic growth is more likely to occur, the longer it is since someone has experienced a bereavement. Both groups were then compared using the Mann Whitney Test.

**Table 2.** To Examine the Relationship Between Length of Time Since Bereavement and Post-Traumatic Growth.

Subscale	More recent loss n = 53	More distant loss n = 47	Mann Whitney
Relating to others	20.45 (7.00)	20.87 (6.61)	z = −.315, p = .753
New possibilities	14.02 (5.74)	14.94 (4.67)	z = −.512, p = .608
Personal strengths	13.13 (3.86)	13.00 (4.06)	z = −.354, p = .724
Spiritual change	6.94 (2.46)	7.30 (2.47)	z = −.780, p = .436
Appreciation of life	8.87 (3.52)	9.32 (2.72)	z = −.559, p = .576
Total PTGI score	63.42 (17.10)	65.42 (17.67)	z = −.380, p = .704

While the scores on 4/5 PTGI subscales were higher in the group who had lost their patent more than five years ago, and their total PTGI scores were higher 65.42 vs 63.42, **none** of these differences was statistically significant.

**Hypothesis 1 is not supported.**

2. **Participants with higher post-traumatic growth will experience significantly lower Adult Attitude towards Grief scores.**

It seems reasonable to hypothesise that bereaved adults with high post-traumatic growth scores on the PTGI will have significantly lower scores on the Adult Attitude Towards Grief scores (see Table 3). This scale measures resilience, being controlled in the face of grief and feeling overwhelmed by grief. Additionally, the scale provides a Vulnerability category score. To test this hypothesis, the sample was divided into a group with low scores on the PTGI (scores from 21 to 68, n = 55), and high scores on the PTGI, (69 to 97, n = 45).

**Table 3.** To Show the Relationship Between Scores on the Post-Traumatic Growth Inventory and the Adult Attitude Towards Grief Scale.

Subscale	Low PTGI scorers n = 55	High PTGI scorers n = 45	Mann Whitney
Resilience	4.69 (sd = 2.81)	4.98 (sd = 2.67)	z = −.401, p = .688
Controlled	7.67 (sd = 2.47)	7.09 (sd = 2.40)	z = −.946, p = .344
Overwhelmed	7.07 (sd = 2.92)	6.71 (sd = 3.14)	z = −.293, p = .770
Total AAG score	19.44 (sd = 4.25)	18.78 (sd = 4.34)	z = −.688, p = .491

On three of the four comparisons the group with the highest Post-Traumatic Growth Inventory scores, had lower, therefore better sores on the Adult Attitude Towards Grief Inventory. None of these differences was significant. When the two groups were compared on all nine individual items on the AAG, there were no significant differences either. Finally, the AAG can be scored at a categorical level as a Vulnerability estimate. People who score more than 24 are said to show High Vulnerability, those who score between 21 and 23 show Medium Vulnerability, and those who score 20 or lower show Low Vulnerability. The groups broke down as follows:

          Low Post Traumatic    High Post Traumatic

          Growth           Growth

Low Vulnerability     36             30

Medium Vulnerability     8               9

High Vulnerability       11              6

These proportions are not significantly different, Chi-square = 1.086, df = 2, p = .582.

**Hypothesis 2 is not supported.**

3. **Participants with higher post-traumatic growth scores on the Post-Traumatic Growth Inventory, will have significantly higher scores on the CABLE Coping Skills Scale.**

It seems reasonable to assume that bereaved adults who have higher levels of Post Traumatic Growth on the PTGI, will have better coping skills than those with low scores on the PTGI (see Table 4). Using the same split in PTGI groups as with the previous measure, the groups were again divided into a High and Low PTGI group. Their scores on the six subscales of the CABLE Coping Skills Scale were compared using the Mann Whitney Test.

**Table 4.** Comparing High Post-Traumatic Growth Scorers Versus Low Post-Traumatic Growth Scorers on the CABLE Coping Skills Scale.

Subscale	Low PTGI scorers n = 55	High PTGI scorers N = 45	Mann Whitney
Help seeking	5.78 (sd = 6.55)	4.04 (sd = 4.45)	z = −1.075, p = .282
Positive outlook	10.53 (sd = 3.87)	12.47 (sd = 4.10)	z = −2.408, p = .016
Spiritual support	9.51 (sd = 3.58)	10.22 (sd = 2.93)	z = −1.160, p = .246
Continuing bonds	9.71 (sd = 4.19)	10.04 (sd = 4.52)	z = −.445, p = .657
Compassionate outreach	6.80 (sd = 2.06)	8.00 (sd = 1.65)	z = −3.187, p = .001
Social support	6.04 (sd = 3.40)	6.44 (sd = 3.38)	z = −.661, p = .509
Total score on CABLE	48.36 (sd = 14.20)	51.22 (sd = 13.30)	z = 977, p = .328

The high PTGI scorers had higher scores on all but one of the seven comparisons, but only two of these were statistically significant differences.

The hypothesis receives only weak support.

## Discussion

The current study examined post traumatic growth and grief amongst bereaving adults following parental death. It proposed three hypotheses. First, that participants whose bereavement, occurred more than five years ago would have significantly higher levels of post-traumatic growth on the PTGI. This was not supported. Second, that participants with higher post-traumatic growth would experience lower Adult Attitude Towards Grief scores. This was not supported. Lastly, that participants with higher levels of post-traumatic grief scores on the PTGI would have significantly higher scores on the CABLE Coping Skills Scale. This received only partial support, with high PTGI scorers having significantly higher scores on the positive outlook and compassionate outreach subscales. Post-traumatic growth did not relate in any predicted way with the other dependent variables in this study.

It is possibly the case that the individuals who took part in the present study may not have been typical of other populations. Yet, in terms of their scores on the post-traumatic growth inventory, they scored significantly higher than a sample of cardiac patients surveyed by Sheikh and Marotta (2005). Scores for the cardiac sample, n = 108, were mean = 56.84, (sd = 24.19), for the current sample, n = 100, the mean score for the PTGI was 64.36 (sd = 16.44), (t test, p = .009). In contrast, comparisons on the CABLE gave the following results. Mean from the present sample = 49.65 (sd = 13.81), in contrast to Crunk (2017), n = 441, mean = 69.62 (sd = 16.15), (t test, p = .001). The current sample would appear to have poorer coping skills. Finally, on the AAG, the current sample averaged 19.14 (sd = 4.28), while the Sim et al. (2014) study found a mean = 22.15 (sd = 4.38), (t test, p = .001). This suggests the present sample was not as distressed as the Sim et al sample. It seems particularly hard to compare scores with different groups on the PTGI, as they may have experienced completely different clinical conditions or possess different characteristics. For instance, while Patrick and Henrie (2016), studied 414 bereaved individuals, they had been bereaved for a period of less than two years, and had even lower scores than the present sample mean = 41.78 (sd = 24.55). It seems reasonable to conclude that the present sample of bereaved adults had high levels of post-traumatic growth, but that these did not relate in any predicted manner with the other dependent variables used in this study. PTGI total score correlated r = .135 with time since parent died, r = .152 with CABLE Total and r -.165 with AAG Total. These are all very low correlations.

### Limitations of the Present Study

The current study was limited to adults above the age of 18. It was restricted to adults who had lost one or both of their parents in the last 10 years only. This study could have been conducted on a broader spectrum of individuals by including those who may have lost one or both of their parents more than 10 years previously. Moreover, only quantitative data were collected in this study to investigate the hypotheses. In depth interviews of the bereaving adults would have provided more insightful information regarding the coping strategies and areas of growth. A sample size of only 100 participants was used in the present study. A larger sample size could have increased the generalizability of the study.

### Implications of the Findings

The findings of the present study have theoretical as well as practical implications. In terms of theory, the current study attempted to fill in a major gap in literature pertaining to post traumatic growth and grief following parental death in adulthood. Most of the research available is focused on the causal effects of parental death on children or adolescents, thus ignoring the impact of parental death on adults. Focusing on bereaving adults thus provided an additional theoretical framework of the process of bereavement and post traumatic growth. There is an assumption that post traumatic growth follows bereavement. Autoethnographic accounts may challenge this assumption and is certainly the case for the two authors of this paper. Author one commented,

Case Vignette: Author 1.

*“Hope is a thing that gives a reason for living. If you lose that reason, you lose hope and you feel you are sinking into a bottomless pit of nothingness which becomes the biggest hindrance to your future growth. A week before my 30^th^ birthday, I met my father and he said that he would surprise me on my 30^th^ and we would celebrate it properly. Little did anyone know, that the surprise on my 30^th^ birthday would be his funeral. I saw him, I felt him, I didn’t touch him though. His lifeless body with an obvious smile on his face was bizarre. Why was he smiling? All the grief theories I had read all my life were just coming true now. If I had to define my feelings in one word then the word has to be NUMB. And I believe that it is the normal reaction to any grief situation. I don’t remember crying much that night. Numbness, shock, disbelief, the classic bereavement theory words. I experienced them all, followed by betrayal. He was just 56 years old, perfectly healthy and active. He had a sudden cardiac arrest and that was it. Two years and I still feel betrayed, hurt and abandoned. I didn’t allow myself to grieve his death though, as I felt I had to bravely perform my role of being the eldest sibling, a daughter and a wife. Maybe it is very early for me to decide whether or not there was any post traumatic growth in my case, as I cannot imagine a person in the world whom I can love as much as I loved my father. As long as I live and as long I celebrate my birthday, I will mourn his absence as I lost a major chunk of myself after losing him.”*

The second author offers the following reflections,

*“Quantitative researchers are meant to be very objective, whereas there is a tradition in qualitative research of researchers declaring their bias. Anyone who has lost a parent whom they were close to, will have had their life thrown into turmoil. I lost my own mother at 16, younger of course than the participants in this study, who all had to be adults. I was the oldest of five children. The single event defined the history of our entire family. Today I just received a text from my mother’s sole surviving sibling, now 85, saying she would be proud that we had all attended a “virtual mass” in Northern Ireland last Sunday (an online service due to the Covid-19 situation). Her own family was defined by the death of her oldest son, one of six, in the same way as our family was defined by the death of our mother. My mother’s death was traumatic. She fell down the stairs and broke her neck, dying on the way to hospital. That was 57 years ago, yet the tears come as I write these few words. Post traumatic growth. No. Life goes on, we adapt. That hole in our heart can never be filled. There is no doubt that post traumatic growth exists, but I don’t think I ever experienced it.”*

In terms of the practical implications of our study, the findings may inform bereavement counsellors and support groups. Bereaving individuals who score higher on the Adult Attitude to Grief Scale (AAG), can be referred to support groups or counsellors for help. People who have been through this trauma can also benefit from understanding the various coping strategies used by bereaving adults. It was found in the present study that crying and praying were the most common and frequently used coping strategies amongst bereaving adults. Other coping strategies reported were sharing grief with family and friends, giving to charity, keeping busy and denial.

### Suggestions for Future Studies

The present research may provide a catalyst for future researchers who might examine the effects of parental death on other variables such as the type of relationship between the deceased parent and the bereaving adults. Furthermore, the personality type of the bereaving adults can also influence the post-traumatic growth and grief that they may experience. These issues may be explored by combining qualitative and quantitative methods. A mixed methods approach towards data collection adds value to research in terms of increasing its validity, by adding a second source for data collection (Hurmerinta-Peltomaki & Nummela, 2006; O’Cathain et al., 2010). Future research will undoubtedly be shaped by the effects of the Covid-19 pandemic, currently faced by the entire world.

## Conclusions

The present study represented an attempt to examine post-traumatic growth and grief in bereaving adults, following the death of their parents in the last 10 years. The authors offered three hypotheses related to post-traumatic growth. These were its relationship with time since parental death; its relationship to levels of grief; and its association with coping skills. Post-traumatic growth showed little relationship with the study dependent variables. Yet, it seemed to be the case that this group of bereaved adults had higher scores on the PTGI, than some comparison groups. Other variables may have had a better relationship with PTGI than the ones studied here, for example, flourishing (Seligman, 2011).It is our contention that post-traumatic growth cannot be assumed after bereavement. Indeed, many people claim never to recover from some deaths, for example parents after the death of a child and in the case of this paper, adult children after the death of a parent. The authors humbly presented their own parental bereavement stories in brief. Neither claimed to have experienced post-traumatic growth. Since this study was conducted, the World is now dealing with the Covid-19 pandemic. It seems likely that the nature of death during the pandemic, with many people dying cut-off from their loved ones in their last days, is more likely to lead to complicated grief reactions, than post-traumatic growth. How will Covid-19 change our theories of bereavement and post-traumatic growth?
